# D-dimer as a Rule-Out for Aortic Dissection

**DOI:** 10.7759/cureus.50170

**Published:** 2023-12-08

**Authors:** Jeremy M Carter, Roshan B Tom, Raheed Sunesra, Nathaniel J Bilby, Blake Mireles, Krishna K Paul, Paul A Koscumb, Mitchell W Cox, Dietrich V Jehle

**Affiliations:** 1 Department of Emergency Medicine, University of Texas Medical Branch at Galveston, Galveston, USA; 2 Department of Vascular Surgery, University of Texas Medical Branch at Galveston, Galveston, USA

**Keywords:** emergency medicine, cardiovascular, dissection of thoracic aorta, d-dimer, acute aortic dissection

## Abstract

Introduction

Acute aortic dissection (AAD) represents a significant diagnostic challenge with a high mortality rate if not treated promptly. This challenge arises from the diverse clinical presentations of AAD, and its symptom overlap with other medical conditions. Although both helical CT and transesophageal echocardiography are reliable diagnostic tools for AAD, they are not feasible for every suspected case. Furthermore, limited research on D-dimer's utility in ruling out AAD has been conducted due to the condition’s rarity.

Methods

This study utilizes the TriNetX database (https://trinetx.com/), encompassing data from 54 healthcare organizations across the United States over the past two decades from 85 million patients. The objective is to evaluate the sensitivity of an elevated D-dimer level in diagnosing AAD across a much larger patient cohort than previously studied.

Results

Retrospectively analyzing this dataset, there were 1,319 patients identified with a confirmed AAD who had undergone D-dimer testing within a day of diagnosis. Of these, 1,252 patients exhibited D-dimer levels exceeding 400 ng/ml while 1,227 had levels surpassing 500 ng/ml. Notably, a D-dimer cutoff of 400 ng/ml demonstrated a sensitivity of 0.949 while a 500 ng/ml cutoff yielded a sensitivity of 0.930.

Conclusion

This large retrospective cohort study demonstrates that a blood D-dimer level is highly sensitive in assaying for AAD. The D-dimer levels analyzed showed a remarkable sensitivity in ruling out AAD, avoiding the need for more invasive testing in low-risk patients.

## Introduction

Acute aortic dissection (AAD) is a relatively rare yet potentially fatal diagnosis. The incidence of AAD is estimated to be about 3.5 per 100,000 [1,2]. The disease process was first described by the autopsy performed on King George II in 1761 [3]. Untreated AAD has an estimated mortality rate of 1% to 2% per hour. With improvements in surgical techniques and medical management, the overall mortality has decreased from 31% to 22% for Type A AADs and has remained at about 13% for Type B dissections [4,5]. Prompt diagnosis decreases mortality but can be difficult due to the variety of presentations, which can imitate more common disease processes such as acute coronary syndrome, pulmonary embolism, renal colic, or stroke. The most common presentation is the acute onset of chest or back pain, which occurs in about 85% of AAD [6]. Timely diagnosis can be further complicated by painless AAD, which occurs in about 6% of patients and presents with syncope, neurologic deficit, or new-onset congestive heart failure. Patients who present without severe chest or back pain have a higher mortality than those with a more classic presentation, due to delays in diagnosis [7]. Classic physical exam findings such as hypertension, diastolic murmur, and widened pulse pressure are frequently absent, contributing to a further delay in the diagnosis with increased mortality [6-8]. Although there is an association between > 20 mmHg asymmetry in upper extremity systolic blood pressure and Type A AAD, this finding frequently occurs in patients without aortic dissection and can be absent in patients with Type A AAD [9,10].

Helical computed tomography (CT) or CT angiography is nearly 100% sensitive and is the most widely used imaging modality for hemodynamically stable patients. Transesophageal echocardiography (TEE) is a viable option and is more widely used for hemodynamically unstable patients. TEE is user-dependent, resulting in higher rates of false positives and negatives with inexperienced operators [6,11]. The increasing use of CT has been a well-documented issue in emergency medicine. Overuse of CT leads to unnecessary costs and increased length of stay. Numerous strategies have been developed to curb the use of CT [12,13]. To reduce the number of unnecessary CT scans, a biomarker with a high sensitivity for AAD would be ideal to facilitate a diagnosis, as this is currently being adopted in some other parts of the world.

We sought to utilize the TriNetX database (https://trinetx.com/) to perform a retrospective analysis to identify the sensitivity of D-dimer in evaluation for AAD. The large number of patients in the US cohort of TriNetX allows our analysis to have greater power than previous studies.

This article was previously presented as a virtual meeting abstract at the 2023 TCEP Research Forum on April 18, 2023.

## Materials and methods

TriNetX is an international health research network providing access to retrospective medical records of approximately 85 million patients within 54 healthcare organizations in the United States. These are predominantly large tertiary care centers and their satellite facilities [14]. Patient data are de-identified and searchable by several specific factors, including diagnoses, procedures, medications, laboratory values, and genomic information (TriNetX). Data were obtained from the United States Collaborative Network database, in which 1,319 patients were identified. Participants included those who were diagnosed with "Dissection of thoracic aorta" (ICD-10: I71.01), as defined by the International Classification of Diseases, Tenth Revision, Procedure Coding System (ICD-10), and who received "Fibrin D-dimer" (LOINC: 48065-7) drawn at the time of diagnosis (one day before or one day after). The study period included the previous 20 years, up to October 16, 2022. Patients were then divided into groups based on a D-dimer concentration ranging from 250 ng/ml to 2,600 ng/ml in 50-100 ng/ml segments. Fifty ng/ml ranges of D-dimer were evaluated for D-dimers less than the normal range of 500 ng/ml and 100 ng/ml ranges for D-dimer over 500 ng/ml. Patients with a "Personal history of other diseases of the circulatory system" (ICD-10: Z86.79), including prior diagnosis of aortic dissection, at least three months before the index event, were excluded from the study.

## Results

**Figure 1:**
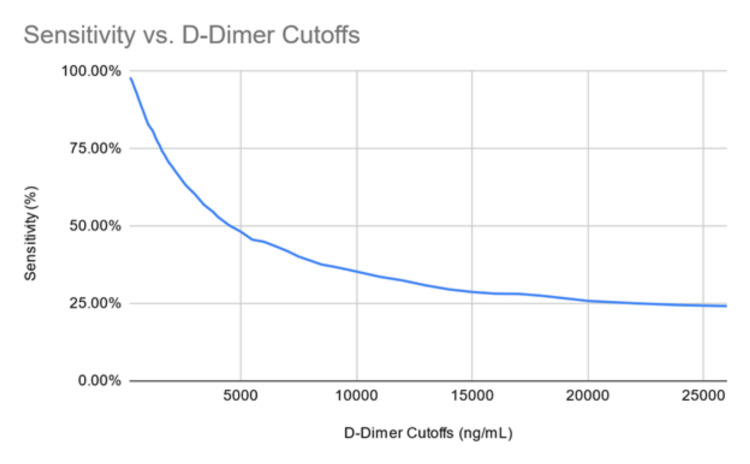
Sensitivity (%) versus D-dimer cut-offs (ng/mL)

We identified 86,341,971 patients in the US cohort of the TriNetX database. There were 1,319 patients with a diagnosis of AAD identified who had a D-dimer drawn one day before or after the diagnosis. Of that total, 1,252 patients had a D-dimer greater than 400 ng/ml, and 1,227 had a D-dimer greater than 500 ng/ml. At cut-off values of 400 ng/ml and 500 ng/ml, the D-dimer has sensitivities of 0.949 and 0.930, respectively. The results were similar at a 93.8% sensitivity for the 500 ng/mL cut-off when we ran the study where D-dimers were measured on the same day as the diagnosis of acute aortic dissection. Figure 1 demonstrates the sensitivities and D-dimer cutoffs graphically.

Using the Explore Cohort feature, baseline demographic data were obtained on age, sex, and race/ethnicity. The mean age of the population was 66 years old. There were 61% male, 39% female, 59% white, 26% black, 5% Hispanic or Latino, 2% Asian, 1% American Indian or Alaskan, 1% Native Hawaiian, and 11% Unknown Race in the group of AADs. Table 1 demonstrates the cohort demographics.

**Table 1:** Cohort demographics (n=1,319) SD, Standard Deviation

Demographics	Mean	±SD
Age	66	±14
	% Cohort	
Sex		
Male	61%	
Female	39%	
Unknown	0%	
Ethnicity		
Not Hispanic or Latino	84%	
Unknown Ethnicity	11%	
Hispanic or Latino	5%	
Race		
White	59%	
Black or African American	26%	
Unknown Race	11%	
Asian	2%	
American Indian or Alaskan Native	1%	
Native Hawaiian or Other Pacific Islander	1%	
Other Race	0%	

## Discussion

Several potential limitations exist. Data regarding Stanford Type A and Stanford Type B aortic dissections were not differentiated. Wang and colleagues demonstrated that the serum D-dimer levels of patients with Stanford A aortic dissection were significantly higher than those with Stanford B aortic dissection at 6 h, 12 h, 24 h, and 72 h after admission. Type B aortic dissections had a D-dimer below 500 ng/mL at 1 hr post-admission [15]. It is possible that an overrepresentation of Type A or Type B aortic dissections could have skewed the data.

This study did not evaluate the potential changes in D-dimer values as the time and disease progressed. Several previous studies have demonstrated that early in the disease course D-dimer values may be below 500 ng/ml and be far above this cutoff late in the course [15,16]. Furthermore, previous studies have shown that D-dimer cutoffs can be increased with age. The practice of age-adjusted D-dimer has been validated for both aortic dissection and pulmonary embolism [17]. This study did not take into account the practice of adjusting D-dimer values for the patient’s age, opting instead to use the same cutoff of 500 ng/mL as in prior studies to facilitate the homogeneity of methodology.

This study also lacks exclusion criteria for multiple normal physiologic processes and disease states that can elevate D-dimer. Age, pregnancy, rheumatologic disease, trauma, and liver disease can all affect the D-dimer level, and adjustment or exclusion of these processes could have changed outcomes.

TriNetX or the use of an observational database for evaluating disease can have several limitations such as selection bias, difficulty of controlling for confounding variables, recorded data quality and missing data, and time point cutoffs not reported. However, these drawbacks are mitigated by the large size of the TriNetX database in question as well as the number of hospitals involved. Furthermore, large observational databases, such as TriNetX, can be used as confirmatory comparisons to the data collected in many smaller studies, as in our case where our data is in line with prior studies on the topic of D-dimer in AAD. Future large, prospective studies are warranted, looking at age-adjusted values that are stratified by type A/B and acute/chronic aortic dissection.

Several previous studies helped establish the cutoff for an elevated D-dimer of 500 ng/ml, which has a pooled sensitivity of about 0.97 to 0.98, with a negative predictive value of 0.96, making it a potentially useful tool to rule out aortic dissection. Lower specificities reported by other studies are secondary to the multiple disease processes that clinically mimic AAD, including myocardial infarction, deep venous thrombosis, and pulmonary embolism, which regularly produce elevated D-dimers [18-20].

Our study, while similarly suggesting that D-dimer is highly sensitive, gives a slightly lower value at 0.93. We suspect that our generated value is likely a slight under-representation. In the TriNetX database, it may be difficult to differentiate AAD from chronic aortic dissection. As a result, cases of chronic aortic dissection could have been included in the AAD group, and this might have decreased the sensitivity of D-dimer in this study compared to other studies.

The primary strength of this retrospective study is its size. The largest previous individual study evaluating D-dimer in AAD included 1,035 patients, of which 233 were positive for dissection [21]. Our trial included 1,319 patients with the diagnosis of AAD. To our knowledge, this study includes 5.6 times more cases of AAD than the largest independent study and 2.8 times the number of patients with AAD than the only meta-analysis addressing this topic to date [19]. Our results are in line with prior studies demonstrating that D-dimer is highly sensitive for AAD and can be useful in aiding in the exclusion of this diagnosis.

Since the selected patient population all had the diagnosis of aortic dissection, we were unable to obtain a calculated specificity of the D-dimer with regard to the diagnosis of AAD. Previous studies have reported lower specificities, ranging from 0.42 to 0.56, consistent with the D-dimer being a nonspecific indicator of inflammation and/or clotting and likely to be elevated in many other processes [16,21].

## Conclusions

This study addresses the complex challenges of identifying AAD by investigating the potential utility of D-dimer as a diagnostic marker. The results highlight that D-dimer levels below the normal threshold demonstrate high sensitivity in effectively ruling out AAD, thereby reducing the need for more invasive diagnostic procedures in patients who are considered low risk. These findings can improve the accuracy when making a diagnosis and streamline clinical decision-making for patients with suspected AAD.
